# A community-based approach to non-communicable chronic disease management within a context of advancing universal health coverage in China: progress and challenges

**DOI:** 10.1186/1471-2458-14-S2-S2

**Published:** 2014-06-20

**Authors:** Nanzi Xiao, Qian Long, Xiaojun Tang, Shenglan Tang

**Affiliations:** 1 School of Public Health and Management, Chongqing Medical University, Chongqing, P.R. China; 2 Global Health Research Center, Duke Kunshan University, Kunshan, P.R.China; 3 Duke Global Health Institute, Duke University, Durham, NC, USA

## Abstract

Paralleled with the rapid socio-economic development and demographic transition, an epidemic of non-communicable chronic diseases (NCDs) has emerged in China over the past three decades, resulting in increased disease and economic burdens. Over the past decade, with a political commitment of implementing universal health coverage, China has strengthened its primary healthcare system and increased investment in public health interventions. A community-based approach to address NCDs has been acknowledged and recognized as one of the most cost-effective solutions. Community-based strategies include: financial and health administrative support; social mobilization; community health education and promotion; and the use of community health centers in NCD detection, diagnosis, treatment, and patient management. Although China has made good progress in developing and implementing these strategies and policies for NCD prevention and control, many challenges remain. There are a lack of appropriately qualified health professionals at grass-roots health facilities; it is difficult to retain professionals at that level; there is insufficient public funding for NCD care and management; and NCD patients are economically burdened due to limited benefit packages covering NCD treatment offered by health insurance schemes. To tackle these challenges we propose developing appropriate human resource policies to attract greater numbers of qualified health professionals at the primary healthcare level; adjusting the service benefit packages to encourage the use of community-based health services; and increase government investment in public health interventions, as well as investing more on health insurance schemes.

## Introduction

Epidemiological, demographic and socio-economic transitions in China over the past few decades have caused a shift from communicable to non-communicable chronic diseases (NCDs) [1]. In 2005, NCDs accounted for an estimated 80% of deaths and 70% of disability-adjusted life-years lost (DALY) in China [2]. In 2010, 34% of Chinese adults over 18 years old (330 million) suffered from hypertension and most were not on appropriate blood pressure control medication [3]. About one-third of Chinese people over 18 years old were overweight, using Body Mass Index (BMI) over 24, and 12% were categorized as obese (BMI≥28) in 2010 [4]. The proportion of overweight adults from 18 to 64 years old increased 15% between 2007 and 2010, while the proportion of obese adults increased by 57% during the same time interval [4,5]. According to the World Health Organization (WHO) Report of the Global Tobacco Epidemic in 2008, over 300 million Chinese men were cigarette smokers and 100 million men currently under the age of 30 years will die from tobacco use if such a smoking pattern continues [6]. The prevalence and burden of NCDs in China will continue to grow with a rapid aging population and continuing changes in socio-economic development and lifestyle choices which affect health. Collectively, these pose great challenges to the improvement of health and healthcare in China.

China had a relatively well functioning health system prior to the economic reform launched in the late 1970s. It had faced numerous problems in financing of, and access to, healthcare during the transformation from planned to market economy in 1980s and 1990s. Since the early 2000s, the Chinese government has renewed its efforts to establish a functioning health system to ensure adequate access to essential healthcare for the vast majority of the Chinese population. Universal health coverage is one important goal; ensuring all Chinese people obtain the essential and affordable health services they need without suffering financial hardship. This requires a robust, efficient, and well-financed health system with sufficient capacity, especially in terms of a well-trained and motivated health workforce. It also requires adequate financial protection for access to medicines, technologies and other services.

With a political commitment to universal health coverage, over the past decade China has made notable progress: more than 92% of the Chinese population is covered by health insurance in 2011[7]; numerous public health interventions launched to tackle both communicable diseases (such as HIV/AIDS, tuberculosis), and NCDs (e.g., hypertension and Diabetes Mellitus (DM)), and adequately funded and implemented community-based health service delivery system being established; the national essential medicine system strengthened to improve rational use of medicines for better quality and cost control, especially at the community level [7,8]; community health centers (CHCs) and community health stations (CHSs) in urban areas; township health centers (THCs) and village health stations (VHSs) in rural areas, empowered to play a key role in improving equitable and affordable access to essential healthcare for the population. Furthermore, the government has developed and implemented a series of measures to enhance the capacity of service provision at these health facilities, including construction and equipment standards for these health facilities, the development of guidelines for general practitioners, and training for primary healthcare providers. There is also an increasing number of primary healthcare facilities at grass-roots which now receive financial supports from both central and local governments [7].

In spite of this progress in strengthening of the health system, significant challenges remain in tackling NCDs in China. With the exception of the Urban Employees’ Basic Medical Insurance (UEBMI), many health insurance schemes in China do not offer comprehensive coverage of health services [7-9]. The service benefit packages often prioritize inpatient services – as per the national guidelines developed by the central government [7,10], but most NCD patients require frequent uses of outpatient services. For example, the New rural Cooperative Medical Scheme (NCMS) and Urban Resident Basic Medical Insurance (URBMI) - jointly financed by the central and province government and with modest premium contribution from individual members - often do not adequately meet the needs of NCD patients. Moreover, well-qualified doctors and nurses in China, like elsewhere in the world, are less likely to work at primary healthcare facilities, which may compromise the quality of care at the community level.

This paper discusses and analyzes the progress and challenges faced when implementing a community-based approach for NCD care and management in the context of advancing universal health coverage in China. First, we discuss the burden of NCDs and challenges in managing these conditions, using hypertension and DM as case studies. Second, we analyze various health policy initiatives related to NCD care and management at the community level, in relation to health financing, and health workforce. Finally, we offer suggestions regarding the effectiveness of a community-based approach addressing NCDs in China.

## Burden of NCDs: challenges for China

Table 1 illustrates the rise of hypertension from 1958 to 2010, based on a series of nationwide epidemiological surveys mainly undertaken by China Center for Disease Control (CDC) [5,11-14]. While these data cannot be directly compared due to differences in study designs and measurements, rising hypertension rates in China are strongly evident, and witnessed in various provinces and cities. For example, Li and colleagues surveyed 16,364 rural residents over 25 years old in Shandong province located in Eastern China in 2007 and reported an age-standardized prevalence rate of hypertension of 37% [15]. In a less developed region Ningxia the prevalence rate was 14% in the period of 2008-2009, reported by another survey [16].

**Table 1:** Prevalence rate of hypertension in China 1958 -2010

Year	Prevalence rate (%)	Data source	Study
1958-1959	5.1	The national sampling survey with adults over 15 years	Chinese guidelines revision committee of prevention and treatment for hypertension, 2006
1979~1980	7.7		
1991	13.6		
2002	18.8	The national multistage and stratified sampling survey with adults over 18 years	Li, 2005
2004	18.1	Multistage random sampling of adults aged at 18~69 years from 79-160 diseases surveillance sites	National center for NCD control and prevention and China CDC, 2009
2007	24.9		National center for NCD control and prevention and China CDC, 2010
2010	33.5		Li, 2012

DM is another growing problem in China (Table 2) [12,17-21]. In 2010 the estimated prevalence of diabetes among a nationally representative sample of Chinese adults was 12% and the estimated prevalence of pre-diabetes was 50% [21].

**Table 2:** Prevalence rates of diabetes in China 1979 -2010

Year	Prevalence rate (%)	Data source	Study
1979	0.7	Random sampling in 14 provinces (cities)	National Diabetes Prevention and Control Cooperative Group, 1981
1994	2.5	Random sampling of adults aged at 24-64 years in 19 provinces (cities)	Pan, 1997
1996	3.2	Stratified and cluster sampling of adults aged at 20~74years in 11 provinces (cities)	Wang, 1998
2000	6.4	Stratified and cluster sampling of adults aged at 35~74years in 10 provinces (cities)	Gu, 2003
2002	2.6	Multistage and stratified cluster sampling of adults over 18 years old in 31provinces	Li, 2005
2010	11.6	Multistage, probability sampling in a nationally representative sample ≥18years old	Xu, 2013

The economic burden caused by both hypertension and DM is heavy. According to the national data, 31.9 billion and 10.7 billion Chinese yuan was spent on hypertension and DM treatment in 2002, which accounted for 5.6% and 1.9% of the total national health expenditure respectively [22]. There was no reliable estimation of the economic burden of hypertension and DM in recent years.

## Strategies and policies for NCD prevention and management in China

Most NCDs are associated with preventable risk factors. Effective control of risk factors could reduce the incidence of heart attack, stroke and DM by at least 80% [23]. The incidence and prevalence of these diseases are closely associated with income level, ecological environment, culture and lifestyle [1]. Recently, central and local governments in China have committed to develop and implement a series of strategies including financial, technical and administrative support for NCD prevention and treatment [24]. Table 3 introduces a number of these strategies and programs with associated indicators being used to assess the effectiveness for these interventions.

**Table 3:** Strategies and programs for NCD prevention and management in China

**Strategies**	**Selected Public Health Programs**	**Selected assessment indicators**
• Financial support from each level government • Advocacy and social mobilization to develop supportive community environment for NCD prevention and control • Community health education and promotion • Clarification of NCD prevention and control responsibility by different health facilities (e.g. CDC, NCD specialist, CHC etc.) • Training health practitioners of CHCs on NCD diagnosis, treatment and rehabilitation services • Comprehensive control for multiple risk factors including health guidance on diet, fitness activity and tobacco control etc. • Promoting NCD patient self-management model and home services by general practitioners	• Population-based public health interventions: - Establishing health file - Providing health education and consultant services - Screening examination • Individual-based health interventions: - Four times follow up visits - Health guidance	• Knowledge of population on NCDs: ≥ 70% • Knowledge on blood pressure: ≥ 70% • Knowledge on blood glucose: ≥ 30% • Registration rates: hypertension and DM ≥ 60% of local prevalence or national average level • Management rate of chronic diseases: hypertension ≥ 35%, DM ≥ 30% • Control rate of chronic diseases: hypertension ≥ 50%, DM ≥ 50%

CHCs: community health centers; CDC: center for disease control; DM: diabetics mellitus; NCD: non-communicable diseases

Primary healthcare facilities at the community level play a key role in the implementation of the strategies and programs. They are mandated to provide essential health services for every resident, especially children, pregnant women, the elderly and patients with NCDs. In 2009, the government allocated 15 Chinese yuan per person per year for the national public health intervention programs; in 2011 the budget increased to 25 Chinese yuan per person per year [7]. In rural areas, 60% of this funding was earmarked for township health centers to implement public health interventions, while the remaining 40% allocated to village health stations [25]. Proportions of the total budget specifically for the management of patients with hypertension and DM was 18% and 7%, respectively [25].

In recent year, China CDC issued the guidelines of NCD prevention and treatment, advocating the integration of primary, secondary and tertiary prevention for hypertension and DM at the primary healthcare level [26,27]. Furthermore, the proposed guidelines tailored interventions targeting three different population groups: 1) the general population; 2) high risk group with characteristics of smoker and obesity, high blood pressure, impaired glucose regulation and high hyperlipidemia; and 3) Hypertension and DM patients. For access of the general population to primary healthcare, standardized personal health profile should be established, containing patient demographic, social characteristics, previous medical records, family medical history, records of physical check-up, and life style, among others. In addition, NCD related health education and promotion services should be provided during outpatient visits to community health centers and stations. Blood pressure should be measured for those over 35 years and older. For the high risk group, annual physical check-up is provided for early detection of NCD patients. For Hypertensive and DM patients, at least four follow-up visits per year can be arranged, with free examinations and tests for blood pressure or glucose. By the end of September 2011, 433 million people have established their personal health record cards, and more than 59 million hypertensive and DM patients are now under the NCD case management system in which community health centers and stations in urban areas and township health centers and village health stations in rural areas are key players for these disease prevention and control activities. Other government strategies for prevention, treatment and management of NCDs included the establishment of three national health insurance schemes to facilitate access to clinical care - the urban employee basic medical insurance (UEBMI), urban resident basic medical insurance (URBMI) and new rural cooperative medical scheme (NCMS). The Chinese Ministry of Health also developed a national essential medicines list and relevant policies which encourage the rational use of medicines at primary health facilities at the community level.

## Challenges in addressing NCDs for China

Despite the good progress made to control and prevent NCDs, great challenges remain in China. One challenge is poor knowledge and low awareness about NCDs, particularly at the poor and rural community level. In 2010, only one quarter of DM patients in rural areas were aware of their illness, compared to 40% among urban patients; One fifth of rural patients and two fifths of urban patients received treatment for diabetes [21]. A significant disparity in awareness of NCDs was also found across different socio-economic development regions [21].

While the primary healthcare facilities at the community level are at the center of NCD prevention and management activities, the quantity and expertise of health professionals at these facilities are far from optimal [28]. The total number of primary healthcare workers in urban areas was about three million in 2011; about three qualified doctors/assistants and 3.32 registered nurses per 1,000 population [29]. In rural areas, the ratio of qualified health workers and registered nurses to 1,000 population was only 1.32 and 0.98, respectively, in the same year [29]. Furthermore, the majority of health professionals in both rural and urban primary healthcare facilities had only 2-3 years of medical education/training, and only 8% of health professionals had a university/college degree. This is due largely to the fact it is difficult to attract new medical and public health graduates, and retain qualified professionals in these facilities. Since the economic reform, the income level of healthcare workers in China has been closely linked with their capacity of revenue generation through user fee charges. The sources of income for most health professionals at the primary healthcare level have often come from drug sales and provision of profitable services. As part of the recent health system reform, the government has now allocated more funds to primary healthcare facilities to encourage the provision of quality services. As a result of the reform, the income of healthcare professionals at this level has no longer been driven by over-provision of drugs and services. The incomes of these doctors and nurses have gradually increased, but still remain relatively low [30]. In addition, opportunities for career development and quality education for the children of health professionals are important factors affecting their preference to work in urban health facilities, instead of rural ones [Liu, personal archive, 2013].

Existing health insurance policies in China also presents great challenges in NCD care and management. The service benefit packages offered by the main health insurance schemes in urban and rural areas differ dramatically. These schemes also tend to prioritize the coverage of inpatient services, while the coverage of outpatient services is either inadequate or lacking. These policies often do not go far enough in meeting the treatment needs of NCD patients in particular [10]. While the above-mentioned three health insurance schemes cover 1.3 billion people - over 90% of the total population in China - the reimbursement rate for inpatient service expenditures by the new rural cooperative medical scheme, for example, ranges between 40- 70% in many regions [7]. The report on chronic disease in China in 2006 shows, average expenses per hospital admission for hypertension and DM was 4,338 Chinese Yuan and 5,276 Chinese Yuan, respectively, accounting for 1.5 times and 1.8 times of average annual per capita income of the rural residents, and for 46% and 56% of average annual per capita income of urban residents, respectively [31]. In addition, the proportion of NCMS reimbursement for outpatient service expense was generally much lower than what was reimbursed for inpatient service expenses. One study conducted in Shandong and Ningxia found only around 10% of outpatient service expenditure reimbursable in rural areas in 2006 [32]. As a result, the economic burden of healthcare expenditure placed on NCD patients was pretty significant that 14-15% of rural households with NCD patients spent more than 40% of their non-food expenditure on chronic disease treatment in 2006 [32], a threshold for catastrophic health expenditure defined by WHO.

## Way forward for a community-based approach to NCD care and control in China

The World Bank (2011) has recently raised serious concerns about epidemics of NCDs in China in its report entitled “Toward a healthy and harmonious life in China: Stemming the rising tide of NCDs”. It has proposed a comprehensive strategy for addressing this growing problem and tackling its challenges. As NCD control requires a continuum of care and extensive social support, a community-based approach has been acknowledged as one of the most cost-effective prevention and control measures [33]. We firmly believe that a community-based approach helps to develop “early detection, early diagnosis and early treatment and systematic patient management”, as well as having the added benefit of building a healthy and friendly community environment through community advocacy and participation, and helping to raise awareness and knowledge of NCDs in the population [33].

For the community-based approach to NCD prevention and control to be effective in China, we offer three recommendations in this paper. First, China needs to develop appropriate human resource policies to attract more qualified health professionals to work at primary health facilities, especially in rural areas. There have been a number of experimental studies on how to retain qualified health professionals in rural or remote areas. Findings emanating from these studies indicate the need to including both financial and non-financial incentives, including policies on increasing income level, supporting medical education, and providing more professional career development opportunities. The government of China needs to seriously consider the development of appropriately tailored human resource policies that can meet the needs of different local contexts.

Second, China needs to adjust the service benefit packages offered by the health insurance schemes to be in favor of the use of community-based health services. China has achieved universal health insurance coverage, but most schemes prioritize the coverage of inpatient care because it was believed that expensive inpatient care was the major reason for pushing people into poverty. However, many studies in China and other countries have identified substantial costs incurred from frequent outpatient visits for NCD treatments. That financial burden has become a major factor resulting in medical impoverishment [10,34]. Therefore, the design of benefit packages should be re-visited and modified, as strong evidence has arisen. A focus on prevention and control of NCDs should also help to further mitigate the medical and social costs of NCDs.

Lastly, China needs to continue to increase its investment in public health intervention programs and financial support to health insurance schemes. Increased funding from both central and local governments needs to be directed to the less developed regions and poorer rural areas to support NCD interventions, and health insurance schemes. In addition, it is equally important to strengthen monitoring of the use of funding for public health interventions, at the primary healthcare community level. Finally, the population, especially the poor, needs to be protected financially from high costs related to NCD treatments.

## Abbreviations

BMI: Body Mass Index; CHCs: CDC: China Center for Disease Control; Community Health Centers; CHSs: Community Health Stations; DALY: Disability-Adjusted Life-Years lost; DM: Diabetes Mellitus; NCDs: Non-Communicable Diseases; NCMS: New rural Cooperative Medical Scheme; THCs: Township Health Centers; UEBMI: Urban Employees Basic Medical Insurance; URBMI: Urban Resident Basic Medical Insurance; VHSs: Village Health Stations; WHO: World Health Organization.

## Competing interests

The authors declare no competing interests.

## Authors' contributions

ST and QL developed the outlines of the paper. NX, together with ST and QL, drafted the paper. XN did literature review. XT participated in the literature review and the paper writing. All the authors commented on and approved the final version of the paper.

## Declarations

The cost of funding for this article has been covered by the authors.

This article has been published as part of *BMC Public Health* Volume 14 Supplement 2, 2014: Responsive health systems: working with the community on control of non-communicable diseases (NCDs). The full contents of the supplement are available online at http://www.biomedcentral.com/bmcpublichealth/supplements/14/S2.
